# What We Learned about the Feasibility of Gene Electrotransfer for Vaccination on a Model of COVID-19 Vaccine

**DOI:** 10.3390/pharmaceutics15071981

**Published:** 2023-07-19

**Authors:** Urska Kamensek, Maja Cemazar, Simona Kranjc Brezar, Tanja Jesenko, Spela Kos, Katarina Znidar, Bostjan Markelc, Ziva Modic, Tilen Komel, Tim Gorse, Eva Rebersek, Helena Jakopic, Gregor Sersa

**Affiliations:** 1 Institute of Oncology Ljubljana, Zaloska Cesta 2, SI-1000 Ljubljana, Slovenia; ukamensek@onko-i.si (U.K.); mcemazar@onko-i.si (M.C.); skranjc@onko-i.si (S.K.B.); tjesenko@onko-i.si (T.J.); skos@onko-i.si (S.K.); kznidar@onko-i.si (K.Z.); bmarkelc@onko-i.si (B.M.); zmodic@onko-i.si (Z.M.); tkomel@onko-i.si (T.K.); 2 Biotechnical Faculty, University of Ljubljana, Jamnikarjeva Ulica 101, SI-1000 Ljubljana, Slovenia; 3 Faculty of Health Sciences, University of Primorska, Polje 42, SI-6310 Izola, Slovenia; 4 Faculty of Medicine, University of Ljubljana, Vrazov Trg 2, SI-1000 Ljubljana, Slovenia; 5 Faculty of Health Sciences, University of Ljubljana, Zdravstvena Pot 5, SI-1000 Ljubljana, Slovenia

**Keywords:** DNA vaccination, gene electrotransfer, COVID-19 vaccine, immunological adjuvant, interleukin 12

## Abstract

DNA vaccination is one of the emerging approaches for a wide range of applications, including prophylactic vaccination against infectious diseases and therapeutic vaccination against cancer. The aim of this study was to evaluate the feasibility of our previously optimized protocols for gene electrotransfer (GET)-mediated delivery of plasmid DNA into skin and muscle tissues on a model of COVID-19 vaccine. Plasmids encoding the SARS-CoV-2 proteins spike (S) and nucleocapsid (N) were used as the antigen source, and a plasmid encoding interleukin 12 (IL-12) was used as an adjuvant. Vaccination was performed in the skin or muscle tissue of C57BL/6J mice on days 0 and 14 (boost). Two weeks after the boost, blood, spleen, and transfected tissues were collected to determine the expression of S, N, IL-12, serum interferon-γ, the induction of antigen-specific IgG antibodies, and cytotoxic T-cells. In accordance with prior in vitro experiments that indicated problems with proper expression of the S protein, vaccination with S did not induce S-specific antibodies, whereas significant induction of N-specific antibodies was detected after vaccination with N. Intramuscular vaccination outperformed skin vaccination and resulted in significant induction of humoral and cell-mediated immunity. Moreover, both boost and adjuvant were found to be redundant for the induction of an immune response. Overall, the study confirmed the feasibility of the GET for DNA vaccination and provided valuable insights into this approach.

## 1. Introduction

The COVID-19 pandemic has enabled widespread authorization and acceptance of nucleic acid-based vaccines, i.e., RNA and DNA vaccines. Although DNA vaccination is defined as either viral or non-viral delivery of DNA-encoded antigens, the term DNA vaccination is commonly used to refer to non-viral delivery, i.e., transfection, and the term vector vaccines to refer to viral delivery, i.e., transduction [1,2,3]. The shared advantage of all nucleic acid vaccines is that they bypass the problems with protein production and purification associated with the traditional protein-based vaccines by using the host cell translational machinery to express protein antigens in their native form [1,2]. Once a given antigen is sequenced, nucleic acid-based vaccines can be designed and produced in a matter of days. After DNA vaccination, antigens are expressed from the transfected or transduced cells or presented in the context of MHC molecules, eliciting both humoral and cellular immune responses [2]. A shared advantage of DNA and RNA vaccines over viral vector vaccines is that pre-existing immunity to the vaccine vector (i.e., virus) is not an issue, enabling repeated dosing [3,4]. While a unique advantage of DNA vaccines over RNA and viral vector vaccines is their relative stability at ambient temperatures, removing the need for a cold chain [5,6].

However, unlike viral vector vaccines, neither RNA nor DNA enters the cells by themselves; hence, their efficacy depends heavily on physical and chemical delivery methods. Developers of RNA vaccines have tackled this issue with a chemical delivery method using lipid nanoparticles [7,8], while for the delivery of DNA, gene electrotransfer (GET) is one of the most widely used and well-established methods [6,9,10,11,12]. It is based on a physical delivery method of electroporation (EP), where a transient increase in the permeability of the cell membrane is achieved by the application of electric pulses [13], therefore removing the need for potentially toxic chemicals such as lipid nanoparticles. As a therapeutic application, GET is currently being tested for cancer gene therapy and DNA vaccination against tumor antigens and infectious diseases like HIV-1, avian flu, and, with the event of the COVID-19 pandemic, also in several preclinical and clinical trials for vaccination against SARS-CoV-2 [10,11,14,15,16,17,18,19,20]. As a vaccination approach, GET has all the above-mentioned advantages of DNA vaccines. Using EP for the delivery of genetically encoded antigens has been shown to have a significant impact on DNA vaccination efficacy and immunogenicity by improving transfection as much as 1000-fold over injection alone [10,21,22,23,24,25]. Additionally, DNA vaccine immunogenicity can easily be further enhanced and modulated by the co-delivery of genetically encoded immune adjuvants, such as interleukin 12 (IL-12) [14,26].

The intramuscular and intradermal routes of administration are the main routes of DNA delivery in both preclinical and clinical trials of DNA vaccines [1,6,15,27,28,29,30,31,32,33]. Skin is our biggest immune organ, crowded with immune cells that are critical to eliciting an immune response, while muscles have a higher capacity for transgene production [34,35,36]. In our previous studies, we have optimized the protocols for the delivery of plasmid DNA both into skin and muscle tissue using GET [29,30].

The aim of this study was to investigate the feasibility of these protocols in the context of vaccination against COVID-19. As the source of antigens, plasmids encoding SARS-CoV-2 spike (S) and nucleocapsid (N) protein were used; additionally, an IL-12-encoding plasmid was used as an immune adjuvant. DNA vaccination, with or without the adjuvant, was compared between skin and muscle tissue based on the induced humoral and cell-mediated immune responses against the expressed antigens. Both skin and muscle vaccination were well tolerated; however, vaccination to muscle resulted in higher expression and induction of immune response. Furthermore, the results indicated that the S-encoding plasmid did not lead to the proper expression of the S antigen and, as a consequence, was not able to induce the humoral immune response. Regardless, S-specific T lymphocytes were induced, highlighting the importance of alternative presentation through the class I pathway for the induction of cell-mediated immune response, which was proven vital for protection against COVID-19. Overall, the study confirmed the feasibility of the GET platform for DNA vaccination against infectious diseases like COVID-19; additionally, it resulted in some valuable findings regarding the presentation of incorrectly expressed S antigen and the role of boost vaccination and immunological adjuvant, which we further discussed in this paper.

## 2. Materials and Methods

### 2.1. Plasmids

Two plasmids encoding the SARS-CoV-2 spike (pS) or nucleocapsid (pN) protein (pUNO1-SARS2-S-d19 (D614G), pUNO1-SARS2-N, InvivoGen, San Diego, CA, USA) were used as the source of antigens. In the in vivo experiments, an IL-12-encoding plasmid (pIL12) pORF mIL-12 (p40::p35) (InvivoGen) was used as an immunological adjuvant. Additionally, an in-house plasmid without a eukaryotic expression cassette pControl [36] was used as a control plasmid to distinguish any nonspecific effects of the plasmid DNA itself. Plasmids were transformed into the *E. coli* JM109 strain using the TransformAid Bacterial Transformation Kit (Thermo Fisher Scientific, Waltham, MA, USA). Plasmid DNA was isolated from the bacterial culture and purified using the EndoFree Plasmid Mega Kit (Qiagen, Hilden, Germany) according to the manufacturer’s instructions. The purity and concentration of the plasmids were measured spectrophotometrically using Epoch microplate spectrophotometer and Take3 microvolume plate (BioTek, Winooski, VT, USA). In addition, concentration and identity were confirmed by restriction analysis on an electrophoretic gel. Final concentrations were fine-tuned according to the measurement with a Qubit 4 fluorometer (Qubit™ dsDNA BR Assay Kit, Thermo Fisher Scientific). For in vitro experiments, plasmid DNA was eluted in the endotoxin-free water supplied with the kit to a concentration of 1 mg/mL. For the in vivo experiments, plasmid DNA was eluted in saline to a concentration of 2.5 mg/mL. Plasmid mixtures were prepared to contain a total of 45 ug of plasmid DNA per 1 vaccination, i.e., 20 µg of S and/or N plasmid, 5 µg of IL-12 plasmid, and pControl summing up to 45 µg, as indicated in Table 1.

### 2.2. Cell Lines

The C2C12 mouse myoblast cell line (American Type Culture Collection, ATCC, Manassas, VA, USA; ATCC^®^ CRL-1772™) and the L929 mouse fibroblast cell line (Merck, Kenilworth, NJ, USA) were cultured in advanced DMEM medium (Gibco, Thermo Fisher Scientific, Waltham, MA, USA), supplemented with 5% fetal bovine serum (FBS, Gibco), GlutaMAX (100×, Gibco), and penicillin–streptomycin (100×, Sigma-Aldrich, Merck, Darmstadt, Germany) grown in a 5% CO_2_ humidified atmosphere at 37 °C. Cells were free of mycoplasma infection, as confirmed by routine testing with a MycoAlertTM PLUS mycoplasma detection kit (Lonza, Basel, Switzerland).

### 2.3. In Vitro GET

A cell suspension with a concentration of 25 × 10^6^ cells/mL was prepared in a cold electroporation buffer (EP buffer; 125 mM sucrose, 10 mM K_2_HPO_4_, 2.5 mM KH_2_PO_4_, 2 mM MgCl_2_ × 6 H_2_O). A total of 40 µL of the cell suspension was mixed with 10 µL of S or N plasmid or nuclease-free water in the vehicle-only control group and pipetted between stainless steel electrodes 2 mm apart, followed by delivery of electric pulses (EP): 8 pulses with a voltage to distance ratio of 1300 V/cm, 100 μs duration, and 5 kHz frequency were used. The procedure was performed in 3 technical replicates (3 electroporations of the same plasmid–cell mixture). Immediately after pulse delivery, the mixture was transferred to a 24-well ultra-low attachment plate, and after 5 min, 1 mL of the appropriate cell culture medium was added. The resulting cell suspension was transferred to a T-75 cell culture flask with a filter cap for evaluation of mRNA and protein expression and to a 96-well plate for evaluation of cell survival. Cells were cultured in a humidified incubator at 37 °C and 5% CO_2_ until further processing.

### 2.4. In Vitro Collection of Samples for mRNA and Protein Analysis

Two days after GET, cell culture medium was collected, centrifuged at 400× *g* for 5 min, divided into aliquots, and stored at −80 °C in Protein LoBind^®^ tubes (Eppendorf, Hamburg, Germany) until the ELISA assay was performed to determine secreted protein expression. The cells were trypsinized (0.25% trypsin-EDTA solution, Gibco, Thermo Fisher Scientific), counted, and divided into two equal fractions. From one fraction, RNA was extracted for subsequent quantification of transgene expression by qRT-PCR (described under Section 2.8). From the other fraction, proteins were extracted for subsequent determination of the non-secreted transgene by ELISA. For protein isolation, cells were centrifuged at 400× *g* for 5 min. To the cell pellet, 150 µL of RIPA buffer (Santa Cruz Biotechnology, Dallas, TX, USA) containing HaltTM protease and phosphatase inhibitor cocktail (Thermo Fisher Scientific) was added. The mixture was incubated on ice for 30 min and mixed thoroughly every 10 min. The mixture was then centrifuged at 12,000× *g* for 15 min. The supernatant was transferred to Protein LoBind^®^ tubes and stored at −80 °C until further use. The details for the ELISA assay and qRT-PCR are given below.

### 2.5. Animals

Female 6–8-week-old C57BL/6NCrl mice were purchased from Charles Rivers (Calco, LC, Italy) and subjected to a quarantine and adaptation period of 1 week. Mice were housed under specific pathogen-free conditions at a temperature of 20–24 °C, relative humidity 55 ± 10%, and a 12 h light–dark cycle. Food and water were provided ad libitum. All procedures were performed in compliance with the guidelines for animal experiments of the EU directive (2010/63/EU) and permission from the Veterinary Administration of the Ministry of Agriculture and the Environment of the Republic of Slovenia (permission no. 822-0001/2021-1). The mice were monitored for weight loss and possible side effects for two weeks after the boost vaccination when the experiment was terminated, and blood, transfected skin and muscle tissue, and spleens were collected from vaccinated mice.

### 2.6. In Vivo GET

**Scheme 1:**
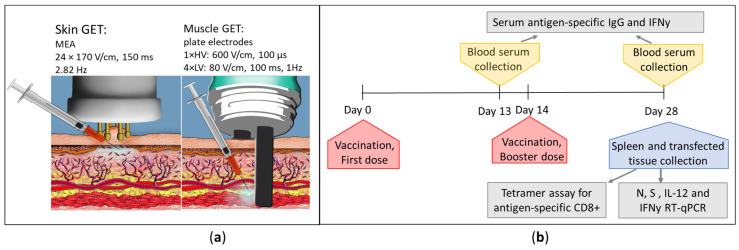
(**a**) Skin and muscle vaccination using GET; (**b**) timeline of the in vivo experiments.

In vivo, vaccination was performed in the skin (right flank) or muscle tissue (right tibialis cranialis muscle) of mice on days 0 and 14 (boost) by GET of the plasmid–DNA mixtures described above. One day prior to the vaccination, the skin over the vaccination area was shaved. On the day of vaccination, mice were anesthetized with isoflurane (1.5% isofluorin, Vetpharma Animal Health, S.L., Barcelona, Spain) to minimize movement during vaccination. The exact treatment area was marked with a permanent marker to ensure that each GET was performed at the same site and to guide the sample collection. Vaccination was performed using previously optimized parameters for transfection of skin and muscle [29,30] (Scheme 1a). Briefly, for skin vaccination, anesthetized mice received a single intradermal injection of 20 µL pf plasmid mixture (described in Section 2.1: Plasmids) in the right flank using a 29 G syringe (Sol-Millennium, Chicago, IL, USA). Immediately after plasmid administration, a contact multielectrode array (MEA, Iskra Medical, Ljubljana Podnart, Slovenia) connected to the Cliniporator™ pulse generator (IGEA s.r.l., Carpi, Italy) was positioned over the injection site, and 24 electric pulses (2 electric pulses between each pair of electrodes) with 170 V/cm amplitude, 150 ms duration, and 2.82 Hz repetition frequency were applied. For muscle vaccination, anesthetized mice received a single muscle injection of 20 µL of the plasmid mixture into the tibialis cranialis muscle using a 29 G syringe. The leg was placed between two flat parallel stainless-steel electrodes with a 6 mm distance between the electrodes, connected to the electric pulse generator (IGEA), and a high-voltage electric pulse with an amplitude per distance of 600 V/cm and a duration of 100 μs and 4 low-voltage electric pulses with an amplitude per distance of 80 V/cm, a 100 ms duration, and a repetition frequency of 1 Hz were applied. Good contact between the electrodes and the skin was ensured by a conductive ultrasound gel (ECO gel for ultrasound, Fiab, Florence, Italy). Two weeks after the initial vaccination, the boost vaccination was performed with the same dose and EP parameters at the same sites (Scheme 1b). Non-treated mice were used as controls.

### 2.7. In Vivo Sample Collection

Blood, spleen, and transfected skin or muscle tissue were collected from vaccinated mice to determine antigen expression and induction of nonspecific and antigen-specific humoral and cell-mediated immune responses (Scheme 1b). Blood was collected at two time points: days 13 and 28 after the first dose of vaccine. Blood was withdrawn from the anesthetized mice by orbital sinus puncture and transferred by capillary tube to the serum-separating tubes (SST Microtainer^®^ blood collection tubes, BD biosciences, Franklin Lakes, NJ, USA). Serum was separated after 30 min incubation at RT by 10 min centrifugation at 1300× *g*. The separated serum was collected in Protein LoBind^®^ tubes (Eppendorf) and stored at −80 °C. Two weeks after the boost (28 days after the first vaccine dose), mice were sacrificed, and transfected skin or muscle tissue and spleen were collected in addition to blood collection. Half of the collected tissue was snap-frozen for RNA isolation, and the other half was fixed in formalin for the histological analysis.

### 2.8. RNA Isolation and Reverse Transcription

For qRT-PCR analysis, RNA was isolated from transfected cells and skin and muscle tissues. Isolation was performed using the peqGOLD Total RNA Kit (VWR Peqlab, Radnor, PA, USA) according to the manufacturer’s instructions, including a DNA digestion step to digest any remaining plasmid DNA that might interfere with the analysis. In the in vitro experiments, cells were lysed with TRK lysis buffer included in the kit, while muscle and skin tissues were ground in liquid nitrogen and lysed with TRIzol™ reagent (Thermo Fischer Scientific). The quantity of isolated RNA was determined using the Qubit 4 fluorometer (Qubit™ RNA Broad Range (BR) Kit, Thermo Fisher Scientific). A total of 500–1000 ng of the isolated RNA was reverse transcribed into cDNA using the SuperScript VILO cDNA synthesis kit (Thermo Fisher Scientific) according to the manufacturer’s instructions in the thermal cycler (Primus 25 advanced^®^ Thermocycler, VWR). The cDNA was diluted to a concentration of 2 ng/μL and stored at −80 °C until further use.

### 2.9. qRT-PCR

Expression of the transfected vaccine antigens (S and N), IL-12, and interferon gamma (IFN-γ) was determined by relative quantification using qRT-PCR and SYBR Green technology. For each reaction, 20 µL of reaction mixture containing PowerUp SYBR Green Master Mix (Thermo Fisher Scientific), 10 ng of the cDNA template, and 200 nM primer pair (Integrated DNA Technologies, IDT, Newark, NJ, USA) were used. The primer pairs used for each target are listed in Table 2. The No Template Control (NTC), containing all reagents except the template (cDNA), was used as a negative control for the qRT-PCR reaction. All samples were run in duplicates. Reactions were run in 96-well PCR plates on a QuantStudio 3 (Thermo Fisher Scientific). Thermal cycling conditions were as follows: 2 min at 50 °C, 2 min at 95 °C, 40 cycles of 15 s at 95 °C, and 1 min at 60 °C, and to determine the melting curve, 15 s at 95 °C, 1 min at 60 °C, and 15 s at 95 °C. The data were analyzed using QuantStudio^tm^ Design & Analysis software v1.4.3. Ct values were determined for each primer set and sample. Expression was expressed as the relative amount of transgene compared to the amount of housekeeping gene.

### 2.10. ELISA

Enzyme-linked immunosorbent assays (ELISAs) were used to determine the expressed secreted and non-secreted S and N proteins in the in vitro experiments, as well as IFN-γ and antigen-specific IgG antibodies in the blood serum after in vivo vaccination. The ELISA kits used are listed in Table 3. Undiluted cell culture medium and isolated proteins from cell lysate after in vitro GET were used to determine the concentration of S and N proteins. Undiluted blood serum was used to determine the concentration of IFN-γ, and 10-fold diluted serum was used to determine the concentration of anti-S and anti-N IgG. Each standard, control, and sample was applied in duplicates, and ELISAs were performed according to the manufacturer’s specific instructions. Within 30 min after completion of the assay, the optical density (OD) for each well was measured at 450 and 570 nm using a spectrophotometer (Cytation 1, BioTek Instruments, Agilent Technologies, Santa Clara, CA, USA). The reading at 570 nm was subtracted from the value at 450 nm to compensate for optical imperfections in the plate. Duplicate readings for each standard, control, and sample were averaged. Protein concentrations were interpolated from standard curves prepared according to the manufacturer’s instructions using the standards supplied with the kits.

### 2.11. Histological Analysis

Formalin-fixed skin and muscle samples were embedded in paraffin, cut into 2 µm sections, and stained with hematoxylin and eosin. Histopathological slides were imaged by NanoZoomer S360MD slide scanner and visualized by NDP.view2 image viewing software U12388-01 (Hamamatsu photonics, Hamamatsu City, Japan). Areas with dense concentration of lymphocytes were selected using the ImageJ [37] selection tool by three independent researchers, and the area under the selection was measured as an indicator of immune cell infiltration.

### 2.12. Tetramer Assay

To evaluate the induction of vaccine-specific CD8+ cells, a tetramer assay was performed on isolated splenocytes. Custom mouse major histocompatibility complex class 1 (MHC I) tetramers (allele: H-2Kb) were ordered for the peptide sequences VNFNFNGL for S and LALLLLDRL for N (Tetramer shop, Kongens Lyngby, Hovedstaden, Denmark). Additionally, anti-Vaccinia virus WR epitope tetramers (H-2Kb/TSYKFESV) were used as a negative control (Tetramer shop). Spleens collected from vaccinated mice were dissected into smaller fragments and subjected to enzymatic digestion in Hanks’ Balanced Salt Solution (with calcium and magnesium; Gibco, Thermo Fisher Scientific) containing 3.2 mg/mL collagenase type 4 (Worthington Biochem, Lakewood, NJ, USA), 0.32 mg/mL Hyaluronidase (Sigma-Aldrich), and 2 U/mL DNase I (Thermo Fisher Scientific) for 30 min with gentle shaking at 37 °C. The digested suspension was then strained through 50 µm strainers (Sysmex, Kobe, Japan) to obtain single cells, centrifuged (5 min, 4 °C, and 400× *g*), and washed in PBS. Red blood cell lysis was performed by incubating the cell suspension in 5 mL 1× Red Blood Cell Lysis Buffer (Biolegend, San Diego, CA, USA) for 5 min. Cells were then resuspended in PBS, and a total of 5 × 10^6^ cells were incubated on ice for 5 min with TruStain FcX PLUS (Biolegend) to block the nonspecific binding of the immunoglobulin to Fc receptors. Cells were then stained with a defined antibody panel: first with tetramers (negative control, anti-Spike, and anti-Nucleocapsid) for 30 min at 37 °C, and then with Fixable Viability Dye eFluor 780 (Thermo Fisher Scientific), anti-CD45, anti-CD8, anti-CD4, and anti-CD3 labeling antibodies for 30 min on ice. The tetramers and antibodies used are listed in Table 4. After incubation, cells were washed twice with PBS and resuspended in IC fixation buffer (Thermo Fisher Scientific). The stained cell suspensions were analyzed using FACSymphony™ A3 flow cytometer (BD Biosciences, Franklin Lakes, NJ, USA). Cells were gated based on forward and side scatter with exclusion of doublets. CD8 T-cells were gated as live, CD45+, CD3+, CD8+ cells. Gates were set based on FMO controls (Supplementary Material Figure S1). Data were analyzed using FlowJo software v10.8.1 (Tree Star Inc., Ashland, OR, USA).

### 2.13. Statistical Analysis

GraphPad Prism 9 (GraphPad software, San Diego, CA, USA) was used for statistical analysis and graphical presentations. The Shapiro–Wilk test was performed to test for data normality. Significance was determined by one-way ANOVA test. Normally distributed data were analyzed with ordinary one-way ANOVA, followed by Tukey’s test for multiple comparisons. Non-normally distributed data were analyzed with a Kruskal–Wallis ANOVA, followed by Dunn’s test for multiple comparisons. A *p* value less than 0.05 was considered statistically significant.

## 3. Results

### 3.1. Expression of Transfected Antigens

**Figure 1:**
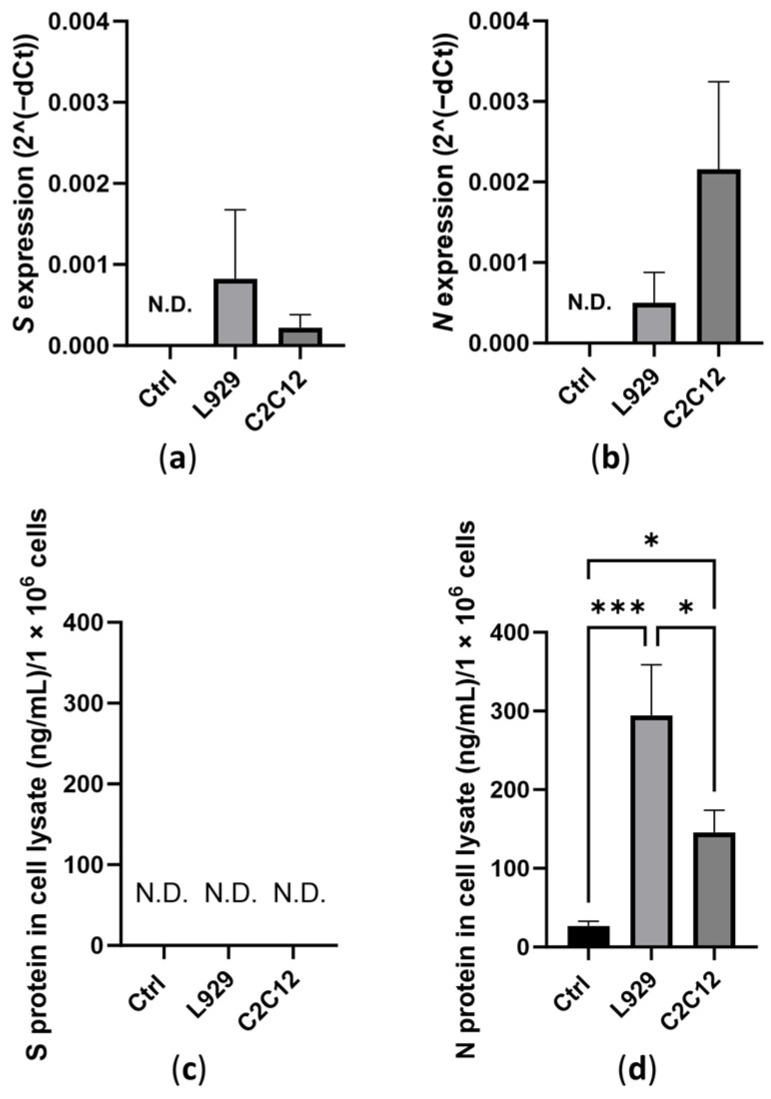
Expression of S and N 2 days after in vitro GET to L929 fibroblasts and C2C12 myoblasts: (**a**) *S* mRNA expression; (**b**) *N* mRNA expression; (**c**) concentration of S protein in cell lysate; and (**d**) concentration of N protein in cell lysate. Graph bars represent the mean with standard error of the mean (SEM) of three independent experiments with 3 technical replicates. Ctrl, non-transfected cells (nuclease-free water + EP); N.D., not detected. One-way ANOVA: *, *p* ≤ 0.05; and ***, *p* ≤ 0.001.

Expression of transfected S and N antigens was determined after both in vitro GET to C2C12 myoblasts and L929 fibroblasts and in vivo GET to muscle and skin tissue. The survival rate after in vitro GET was approximately 80% (Supplementary Material Figure S2a,b), and the expression of S and N mRNA was detected in both cell lines (Figure 1a,b). The production of N was further confirmed at the protein level (Figure 1c,d), whereas S was not detectable in either the cell medium or cell lysate with the available ELISA kits (Supplementary Material Figure S2c,d).

**Figure 2:**
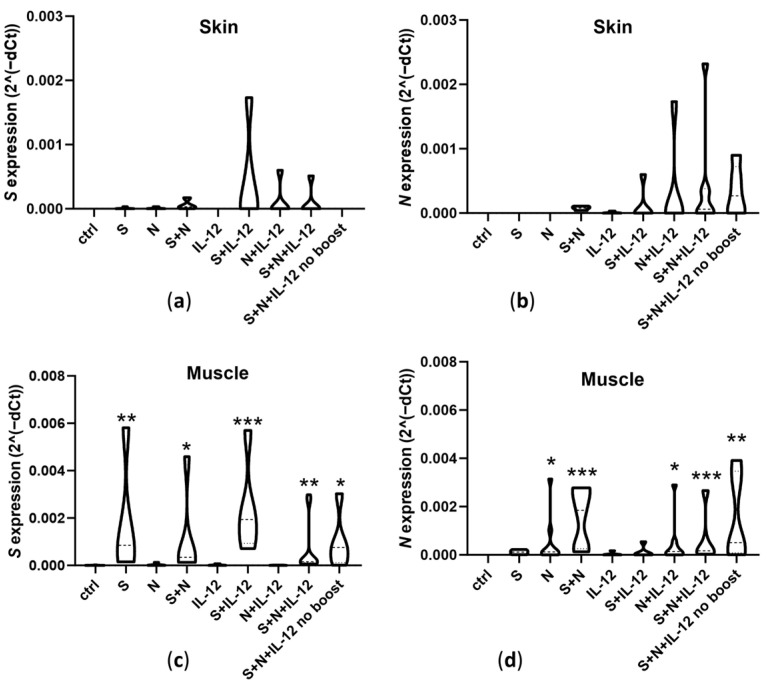
Expression of N and S in skin and muscle tissue 28 days after the first vaccine dose (in vivo GET): (**a**) *S* mRNA expression after skin transfection; (**b**) *N* mRNA expression after skin transfection; (**c**) *S* mRNA expression after muscle transfection; and (**d**) *N* mRNA expression after muscle transfection. Results are presented as violin plots showing data distribution. Total of 5–10 animals per group. Ctrl, non-treated mice. One-way ANOVA vs. Ctrl: *, *p* ≤ 0.05; **, *p* ≤ 0.01; and ***, *p* ≤ 0.001.

Expression from both transfected plasmids at the mRNA level was also detected after in vivo transfection of skin tissue, although levels were too low to be statistically significant (Figure 2a,b). Transfection of the muscle tissue, however, resulted in significant expression from both S and N plasmids and also their combination (Figure 2c,d).

### 3.2. Expression of Il-12 and Ifn-γ and Serum IFN-γ Concentrations

**Figure 3:**
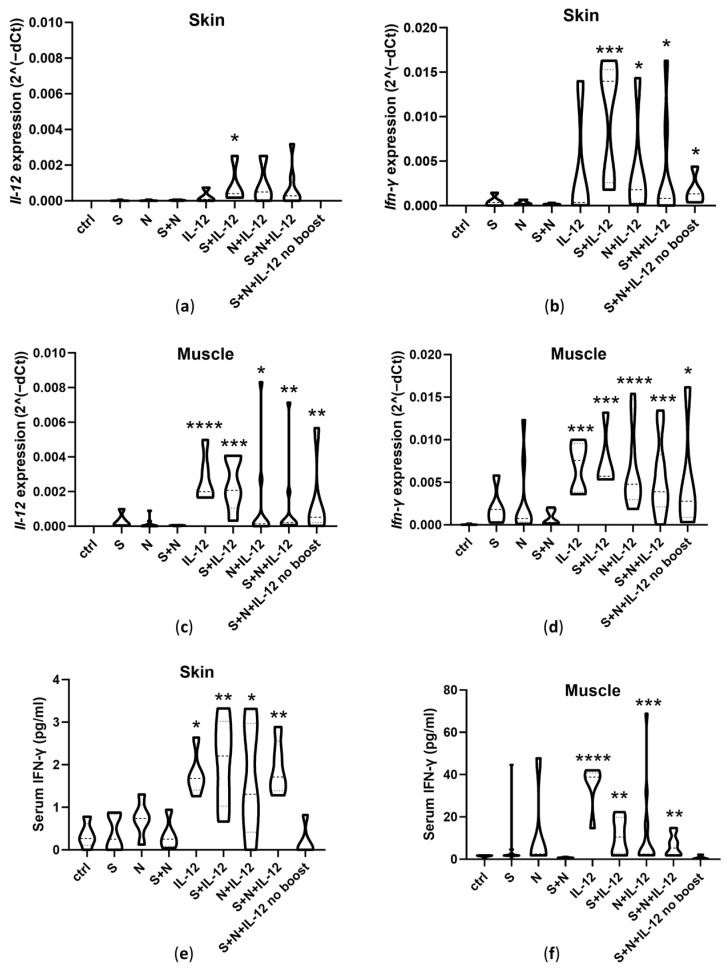
Expression of *Il-12* and *Ifn-γ* in skin and muscle tissue and serum IFN-γ concentrations 28 days after the first vaccine dose (in vivo GET): (**a**) *Il-12* mRNA expression after skin transfection; (**b**) *Ifn-γ* mRNA expression after skin transfection; (**c**) *Il-12* mRNA expression after muscle transfection; (**d**) *Ifn-γ* mRNA expression after muscle transfection; (**e**) serum IFN-γ protein concentrations after skin transfection; and (**f**) serum IFN-γ protein concentrations after muscle transfection. Results are presented as violin plots showing data distribution. Total of 5–10 animals per group. Ctrl, non-treated mice. One-way ANOVA vs. Ctrl: *, *p* ≤ 0.05; **, *p* ≤ 0.01; ***, *p* ≤ 0.001; and ****, *p* ≤ 0.0001.

In addition to the expression of the transfected antigens, expression of IL-12 and its downstream cytokine IFN-γ was also determined in the transfected tissues. In the skin, expression of IL-12 was significantly increased only in the group that received pIL-12 together with the pS, whereas expression of IFN-γ was significantly increased in all groups that received pIL-12 in combination with either of the plasmids or a combination of both plasmids (Figure 3a,b). In muscle, the expression of both IL-12 and IFN-γ was increased in all groups that received pIL-12, either alone or in combination with other plasmids (Figure 3c,d). IFN-γ protein was additionally measured in serum. The result confirmed its increase in all groups receiving pIL-12 alone or in combination with other plasmids both after skin and muscle vaccination (Figure 3e,f).

### 3.3. Histopathological Changes at the Vaccination Site and Mice Well-Being

**Figure 4:**
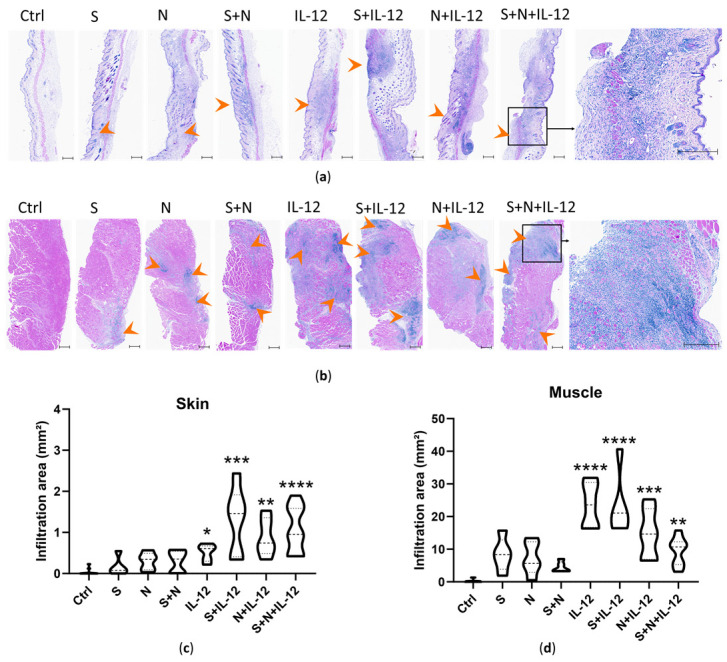
Immune infiltration at the vaccination sites 28 days after the first vaccine dose: (**a**) hematoxylin and eosin stained skin tissue slides; (**b**) hematoxylin and eosin stained muscle tissue slides; (**c**) infiltration area in the skin samples; and (**d**) infiltration area in the muscle samples. Arrows indicate immune infiltration. Scale bar, 300 nm. Results are presented as violin plots showing data distribution. Total of 5–10 animals per group. Ctrl, non-treated mice. One-way ANOVA vs. Ctrl: *, *p* ≤ 0.05; **, *p* ≤ 0.01; ***, *p* ≤ 0.001; and ****, *p* ≤ 0.0001.

Increased infiltration of lymphocytes at the site of vaccination could be seen on the histological slides in both skin and, especially, muscle tissue (Figure 4a,b). The infiltrated area was significantly larger when pIL-12 was used as an adjuvant (Figure 4c,d). Apart from microscopical local inflammation at the site of vaccination, no side effects or mortality was observed, and no signs of toxicity were evident.

### 3.4. Induction of Humoral Immunity

**Figure 5:**
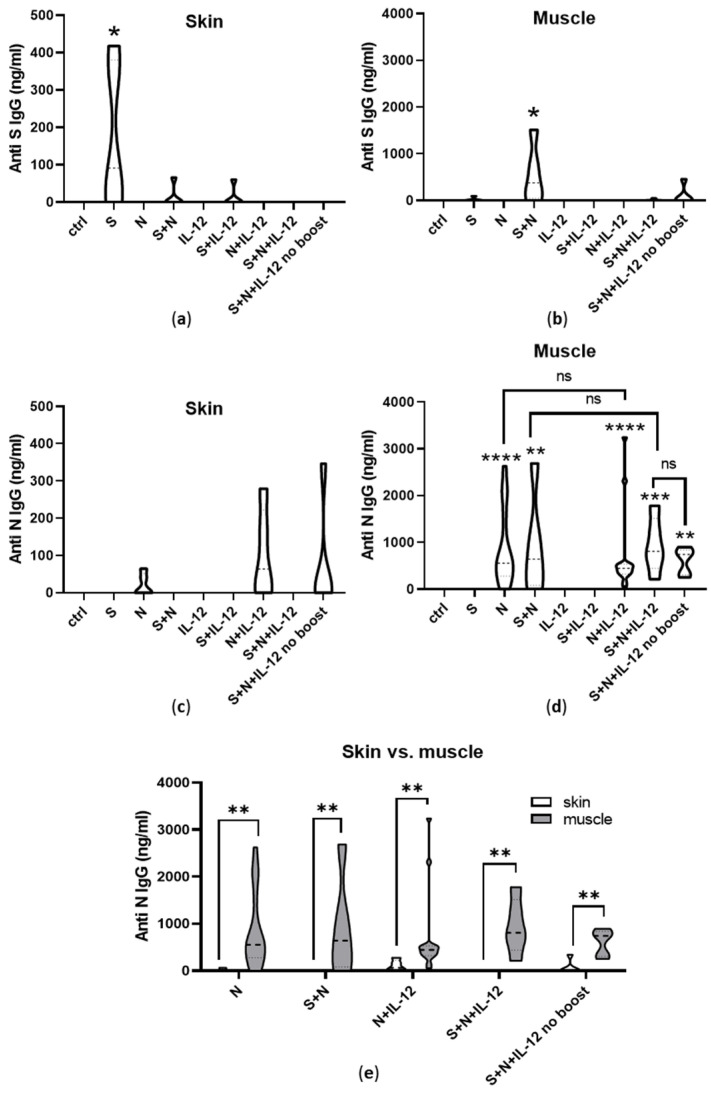
Induction of specific IgG antibodies against the transfected S and N antigens in the blood serum of the vaccinated mice 28 days after the first vaccine dose: (**a**) concentration of anti-S IgG antibodies after skin vaccination; (**b**) concentration of anti-S IgG antibodies after muscle vaccination; (**c**) concentration of anti-N IgG antibodies after skin vaccination; (**d**) concentration of anti-N IgG antibodies after muscle vaccination; and (**e**) comparison of induction of anti-N antibodies between skin and muscle vaccination. Results are presented as violin plots showing data distribution. Total of 5–10 animals per group. Ctrl, non-treated mice. One-way ANOVA vs. Ctrl or between indicated groups in graph e: *, *p* ≤ 0.05; **, *p* ≤ 0.01; ***, *p* ≤ 0.001; and ****, *p* ≤ 0.0001. One-way ANOVA vs. all groups: ns, *p* ≥ 0.05.

The formation of specific IgG antibodies against the transfected S and N antigens was measured in the blood serum of the vaccinated mice on days 13 and 28 after the first dose of vaccine. At day 13, the levels of N- and S-specific antibodies were not significantly elevated, except for anti-N antibodies after muscle vaccination in the S + N and N + IL-12 groups (Supplementary Material Figure S3a–d). After 28 days, vaccination with S still did not result in a general induction of S-specific antibodies (Figure 5a,b). However, a few mice did develop antibodies against the S antigen after both skin vaccination (5 out of 25 mice received the pS plasmid: 3 in the S group, 1 in the S + N group, and 1 in the S + IL-12 group) and muscle vaccination (8 out of 25 mice: 1 in the S group, 4 in the S + N group, 1 in the S + N + IL-12 group, and 2 in the S + N + IL-12 no boost group). Vaccination with N, however, resulted in the induction of N-specific antibodies in 28% of mice vaccinated in the skin (7 out of 25 that received the pN plasmid) and in 98% of mice vaccinated in the muscle (44 out of 45) (Figure 5c,d). Induction of humoral immune response was not statistically higher when pIL-12 was used as an immunologic adjuvant. Also, antibody levels in the boosted group were not statistically higher compared to the group receiving only the first dose. Overall, the induction of anti-N antibodies was significantly higher after muscle vaccination in all groups tested (Figure 5e).

### 3.5. Induction of Cell-Mediated Immunity

**Figure 6:**
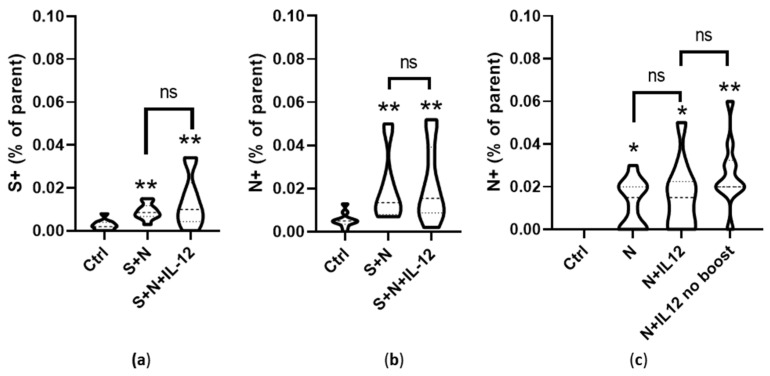
Induction of antigen-specific cytotoxic T-cells against the transfected S and N antigens in the spleens of the vaccinated mice 28 days after the first vaccine dose: (**a**) percentage of S-specific cytotoxic T-cells after vaccination with the mixture of pS and pN plasmids; (**b**) percentage of N-specific cytotoxic T-cells after vaccination with the mixture of pS and pN plasmids; and (**c**) percentage of N-specific cytotoxic T-cells after vaccination with the N plasmid. Results are presented as violin plots showing data distribution. Total of 5-10 animals per group. Ctrl, non-treated mice. One-way ANOVA vs. Ctrl: *, *p* ≤ 0.05; **, *p* ≤ 0.01. One-way ANOVA vs. all groups: ns, *p* ≥ 0.05.

After vaccination in the muscle tissue, we also performed tests for cell-mediated immunity by determining antigen-specific cytotoxic T-cells against the transfected S and N antigens in the spleens of the vaccinated mice. The first experiment was performed on main therapeutic groups using both plasmids, and the second experiment was performed using only the plasmid for N. The results of the first experiment confirmed that vaccination led to the induction of N as well as S-specific cytotoxic T-cells (Figure 6a,b). The second experiment confirmed the induction of N-specific antibodies also when the pN plasmid was used alone and in combination with IL-12 (Figure 6c). Again, the addition of the adjuvant did not lead to a significantly higher amount of N-specific T-cells, and the induction was also not significantly lower without the boost.

## 4. Discussion

One of the positive impacts of the COVID-19 pandemic has been accelerated research of DNA vaccines for infectious diseases and also for other applications. In our group, we are exploring GET as a non-viral gene therapy approach for cancer immunotherapy and vaccination. In the current study, we aimed to evaluate the feasibility of our previously optimized protocols for GET-mediated delivery of plasmid DNA into skin and muscle tissues on a model of the COVID-19 vaccine.

As a source of antigens, we used available commercial plasmids encoding the SARS-CoV-2 proteins spike (S) and nucleocapsid (N). The S protein is the main antigen used in preclinical and clinical trials of COVID-19 DNA vaccines, while the nucleocapsid (N) protein is yet another interesting antigen candidate that has been tested in fewer studies [22,37]. In our study, the expression from transfected S- and N-encoding plasmids on mRNA was detected after both in vitro and in vivo GET (Figure 1). For the in vitro expression tests, we used mouse myoblast and fibroblast cell lines to model for muscle and skin vaccination. After GET, we confirmed the expression from both transfected plasmids in both cell lines. While production of N was also confirmed at the protein level, S could not be detected in either cell medium or cell lysate. This indicated problems with its proper folding and/or expression on the cell membrane [38]. As a complex transmembrane protein, S could be harder to express [39,40]. We assume that the plasmid we used resulted in the expression of a misfolded protein that could not be detected by available ELISA assays. Namely, we used the first-generation spike-encoding plasmid pUNO1-SARS2-S-d19 (D614G), which encodes the SARS-CoV-2 spike G614 wild-type variant, including the receptor binding domain, with a removed ER retention signal (∆19). The sequence includes a functional furin cleavage site, which was inactivated in later versions of the plasmid to yield a prefusion-stabilized protein [41].

At the mRNA level, expression from both plasmids was also detected in vivo after both skin and muscle vaccination. However, the transfection of muscle tissue resulted in a much higher expression, confirming the higher capacity of muscle for transgene production. In vivo, we also evaluated the expression of IL-12, which we added to the vaccine as a plasmid-encoded adjuvant, and also its downstream cytokine IFN-γ (Figure 2). Expression of both was increased in all groups receiving pIL-12 either alone or in combination with other plasmids. IFN-γ protein was additionally measured in serum as a measure of a general induction of both innate and adaptive immune responses [42]. The result confirmed its increase in all groups receiving pIL-12 in combination with one of the plasmids or their combination. This was an expected result, as IFN-γ can be induced by transfected IL-12, as well as type I interferons [43], which are induced by the introduction of plasmid DNA that is recognized by endosomal or cytosolic DNA sensors [44,45]. Additionally, once antigen-specific immunity develops, IFN-γ is produced by antigen-stimulated lymphocytes [42]. The induction of IFN-γ in serum was much more pronounced after muscle vaccination, consistent with the higher expression capacity of muscle tissue mentioned earlier. Furthermore, although muscle tissue is normally poor in immune cells, intramuscular GET has been reported to induce local and transient muscle damage, promoting the recruitment of inflammatory cells and cytokine production at the application site [35,46]. This was also confirmed in our study, as dense infiltration of immune cells was seen at the vaccination site (Figure 3). In the case of DNA vaccination, this is actually considered a desirable reaction to induce an immune response against the target antigen.

Thus, in terms of expression and general induction of the immune system, muscle vaccination outperformed skin vaccination. This was to be expected due to the higher capacity for transgene production and local inflammation caused by EP and plasmid DNA introduction, causing infiltration of immune cells. The skin, on the other hand, has an intrinsically higher concentration of immune cells, including antigen-presenting cells, which could be utilized to induce immune responses during intradermal vaccination [34]. Therefore, our next question was, which route results in a stronger induction of specific immune responses against the transfected antigens? First, we investigated the induction of humoral immunity, i.e., specific IgG antibodies against the transfected S and N antigens (Figure 4). As expected from the expression studies that indicated problems with the proper expression of the S protein, vaccination with S did not lead to a general induction of S-specific antibodies. In contrast, vaccination with N led to the induction of N-specific antibodies in ~ one-quarter of the skin-vaccinated mice and in almost all muscle-vaccinated mice. Moreover, the amount of antigen-specific IgG was significantly higher after muscle vaccination in all groups tested. Therefore, in our study, the muscle route also proved to be superior in terms of the induction of humoral immunity.

We proceeded to examine the induction of cell-mediated responses (Figure 5). Because intramuscular vaccination proved superior to skin vaccination in terms of expression, general induction of immune response, as well as induction of humoral immunity, we assessed the cell-mediated responses only after muscle vaccination, also to comply with the 3R ethical rule for animal testing [47]. In addition, intramuscular DNA vaccination has already been shown to elicit stronger T-cell responses than intradermal DNA vaccination in several earlier studies [10]. Indeed, our results confirmed that muscle vaccination leads to the induction of specific cytotoxic T-cells. Cell-mediated immunity is particularly important to fight viral infections and also cancer [48]. For the resolution of SARS-CoV-2 infection specifically, T-cell responses have been proven crucial, as antibodies are not detected in all naturally infected individuals, and antibodies also tend to fade very rapidly after infection [49]. Therefore, in this scenario, the development of vaccine approaches that can induce both arms of immunity is preferred. DNA vaccines can certainly accomplish this, which was also demonstrated in our study.

Cell-mediated immunity can also be enhanced by targeting antigens for cytosolic degradation [10,38,50]. Unexpectedly, this proved noteworthy in our study. Namely, although we could not detect S protein, and consequently anti-S antibodies, probably because the protein was misfolded, we still detected a significant induction of S-specific cytotoxic T-cells. The induction of cellular, but not humoral, immune response could be explained by the alternative presentation of S epitopes via the MHC I on the transfected cells. Namely, the transfected antigens, when expressed as foreign proteins, are at least partially directed to the proteasome, similar to what happens after viral infection. In the proteasome, foreign or misfolded proteins are degraded to epitope peptides, which are then presented by the MHC I, inducing the activation of specific T-cells [50,51]. This mechanism is part of the cell’s inherent ability to present foreign proteins that evolved as a natural defense mechanism against viral infections and is now proving useful for enhancing T-cell responses against antigens delivered with DNA vaccines.

The next, most common way to direct immune responses toward cell-mediated immunity is by using immunoadjuvants that push the immune response toward the Th1 type [52]. In the case of DNA vaccines, this is relatively straightforward because DNA-encoded cytokine adjuvants can be easily co-delivered with DNA-encoded antigens [10]. Co-delivery of plasmid-encoded IL-12 is particularly widely utilized [26,53]. The importance of IL-12 for the induction of cell-mediated immunity and enhancement of cytotoxic T-cell activity is well known [54]. In our past studies, we successfully used this cytokine as an adjuvant to in situ and tumor cell-based cancer vaccines [55,56,57,58]. In the last experiment of the current study, we tested the role of IL-12 adjuvant for the induction of cell-mediated immunity by using only the plasmid for N with or without IL-12 adjuvant. Interestingly, the addition of IL-12 did not significantly increase the number of N-specific T-cells. Similarly, in terms of humoral immunity, induction was not statistically higher when IL-12 was used as an adjuvant. Apparently, the adjuvant effects of EP and plasmid DNA mentioned earlier might be sufficient to induce both humoral and cell-mediated immunity, so the addition of an immunological adjuvant seems to be redundant for vaccination with GET. This could significantly reduce the time and cost of DNA vaccine development by avoiding the additional production of plasmids and, more importantly, their preclinical toxicological evaluation [59].

Another interesting and unexpected finding was that our results also did not confirm the need for boost vaccination. Indeed, both the antibody levels as well as numbers of antigen-specific T-cells in the group receiving only the first dose of vaccine were comparable to those in the boosted group; however, more experimental groups are needed to confirm this statement. Although DNA vaccination allows repeated administration, unlike vaccination with viral vectors, our results suggest that this may not be necessary. This is probably because the expression of antigens from transfected plasmids is perpetual enough to elicit an immune response without the boost. Namely, expression after GET, especially in muscle tissue, can persist for months [1,29], which is longer compared to short-lived expression using mRNA vaccines [5]. Therefore, using the GET platform for vaccination promises no or fewer boosts and a longer period between boosts.

## 5. Conclusions

DNA vaccination is a promising approach for the treatment and prevention of various types of cancer and infectious diseases. Using SARS-CoV-2 S antigens as a model, we confirmed the feasibility of the GET platform for DNA vaccination. We showed that vaccination in muscle outperforms vaccination in the skin in terms of expression, general induction of immune response, and induction of humoral and cell-mediated immunity. Due to the problems with the detection of S protein, which could be considered one of the limitations of the current study, we also touched on the importance of proper antigen expression and presentation for the desired immune response. In addition, we showed that both immunological adjuvant and boost vaccination may be redundant for the induction of an immune response after vaccination with GET. We hope that these and other similar studies will promote the widespread adoption of GET as a vaccination method against emerging infectious diseases and, most importantly, solidify its role in therapeutic vaccination for cancer treatment. Namely, we believe that DNA vaccination with GET is even more suitable for treating cancer than it is as a prophylactic measure to be given to a healthy population.

## Figures and Tables

**Table 1:** Plasmid mixtures for in vivo experiments.

Groups	pS (µg)	pN (µg)	pIL-12 (µg)	pControl (µg)	Sum (µg)
Ctrl	0	0	0	0	0
S	20	0	0	25	45
N	0	20	0	25	45
S + N	20	20	0	5	45
IL-12	0	0	5	40	45
S + IL-12	20	0	5	20	45
N + IL-12	0	20	5	20	45
N + IL-12 (no boost)	0	20	5	20	45
S + N + IL-12	20	20	5	0	45
S + N + IL-12 (no boost)	20	20	5	0	45

**Table 2:** qRT-PCR primers.

Target	Primer	Sequence
Nucleocapsid (SARS-CoV-2)	N-F	CTCACTCAACATGGCAAGGA
	N-R	GTCTGGTAGCTCTTCGGTAGTA
Spike (SARS-CoV-2)	S-F	GTGCAGGTATATGCGCTAGTT
	S-R	GCACCAAGTGACATAGTGTAGG
Interleukin 12 (mouse)	IL-12-F	CGGCAGCAGATTAAATATGAG
	IL-12_R	GAGTTCTTCAAAGGCTTCATC
Interferon gamma (mouse)	IFN-γ-F	CACACTGCATCTTGGCTTTG
	IFN-γ-R	GCTGTTGCTGAAGAAGGTAGTA
Beta-actin (mouse)	Actb-F	GAAGTGTGACGTTGACATCC
	Actb-R	ACTCATCGTACTCCTGCTTG
Glyceraldehyde 3-phosphate dehydrogenase (mouse)	Gapdh-F	TTCACCACCATGGAGAAGGC
	Gapdh-R	GGCATGGACTGTGGTCATGA

**Table 3:** ELISA kits.

Target	ELISA Kit	Provider
N-Protein	COVID-19 N-Protein ELISA Kit	Abcam, Cambridge, UK
S-Protein	COVID-19 Spike Protein ELISA Kit	Abcam
S-Protein	GENLISA™ Human SARS-CoV-2 (COVID-19) Spike Protein S1 Antigen Quantitative ELISA kit	Krishgen BioSystems, Mumbai, India
Mouse IFN-gamma	Mouse IFN-gamma Quantikine ELISA Kit	Abcam
Anti-S mouse IgG	GENLISA™ Mouse Anti-SARS-CoV-2 (COVID-19) IgG Antibody to Spike S1 + S2 ECD Protein Quantitative TITRATION ELISA	Krishgen BioSystems
Anti-N mouse IgG	GENLISA™ Mouse Anti-SARS-CoV-2 (COVID-19) IgG Antibody to Nucleocapsid Protein Quantitative TITRATION ELISA	Krishgen BioSystems

**Table 4:** Tetramers and antibodies.

Tetramer/Antibody	Supplier	Catalog Number	Conjugate	Dilution
Anti S tetramer H-2Kb/VNFNFNGL	Tetramer shop	Custom	BV786	1:20
Anti-N tetramer H-2Kb/LALLLLDRL	Tetramer shop	Custom	BUV395	1:20
Anti-Vaccinia tetramer H-2Kb/TSYKFESV	Tetramer shop	MHC-LC558	APC	1:20
Fixable Viability Dye	Thermo Fisher Scientific	65-0865-14	eFluor 780	1:1000
CD45 Monoclonal Antibody (30-F11)	Thermo Fisher Scientific	56-0451-82	Alexa Fluor 700	1:66
CD8a Monoclonal Antibody (53-6.7)	Thermo Fisher Scientific	53-0081-80	Alexa Fluor 488	1:83
CD4 Monoclonal Antibody (RM4-5)	Thermo Fisher Scientific	45-0042-80	PerCP-Cyanine5.5	1:83
CD3 Monoclonal Antibody (17A2)	Biolegend	100234	Brilliant Violet 510	1:50

## Data Availability

The data presented in this study are available on request from the corresponding author.

## Supplementary Materials

The following supporting information can be downloaded at https://www.mdpi.com/article/10.3390/pharmaceutics15071981/s1. Figure S1: Flow cytometry gating strategy; Figure S2: In vitro survival and cell-medium S and N protein concentration after vitro GET to L929 fibroblasts and C2C12 myoblasts; Figure S3: Induction of specific IgG antibodies against the transfected S and N antigens in the blood serum of the vaccinated mice 13 days after the first vaccine dose.
