# Supplementary material for: What We Learned about the Feasibility of Gene Electrotransfer for Vaccination on a Model of COVID-19 Vaccine
Source: Pharmaceutics. 2023 Jul 19;15(7):1981. doi: 10.3390/pharmaceutics15071981 (PMC10385748; doi:10.3390/pharmaceutics15071981)
Supplement: Supplementary file 1 [file pharmaceutics-15-01981-s001.zip › pharmaceutics-2456907-supplementary.pdf]

What We Learned about the Feasibility of Gene Electrotransfer for Vaccination on a Model of COVID-19 Vaccine

Supplementary data

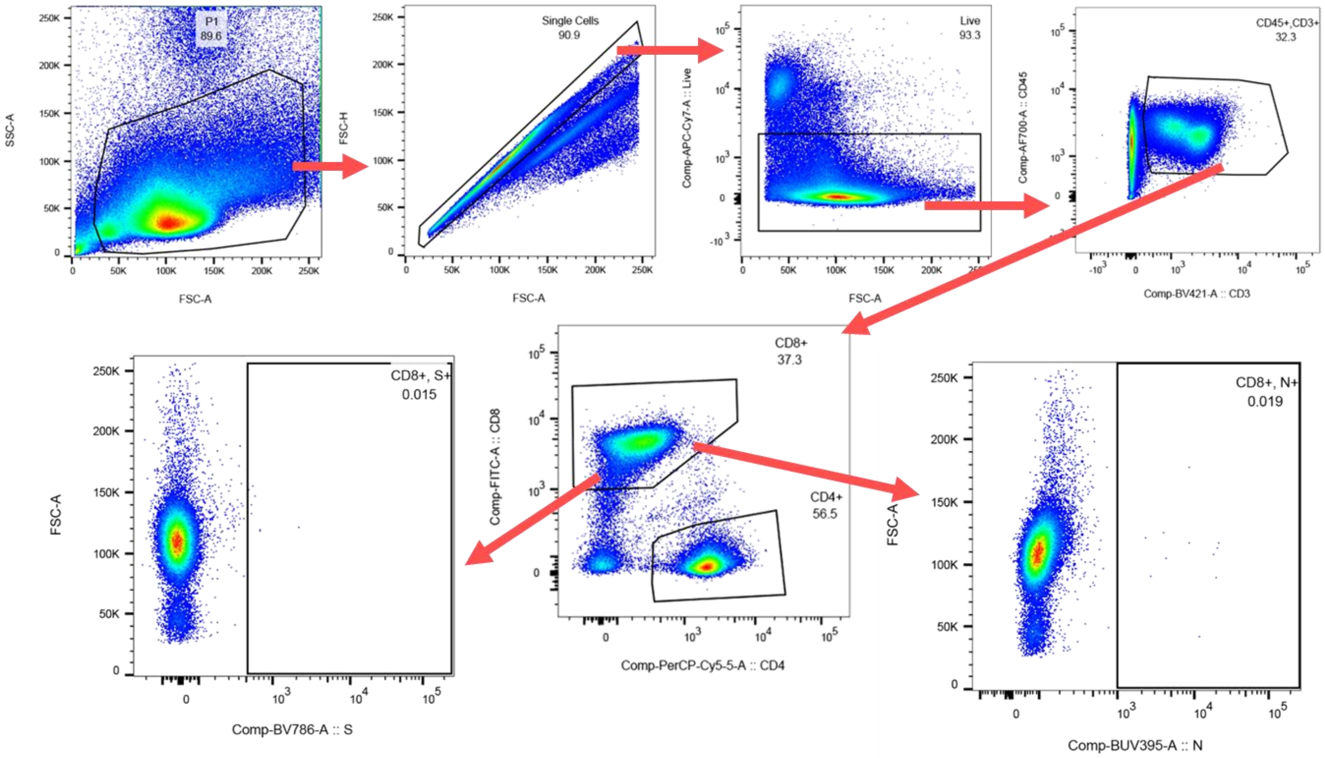

Supplementary Figure S1. Flow cytometry gating strategy.

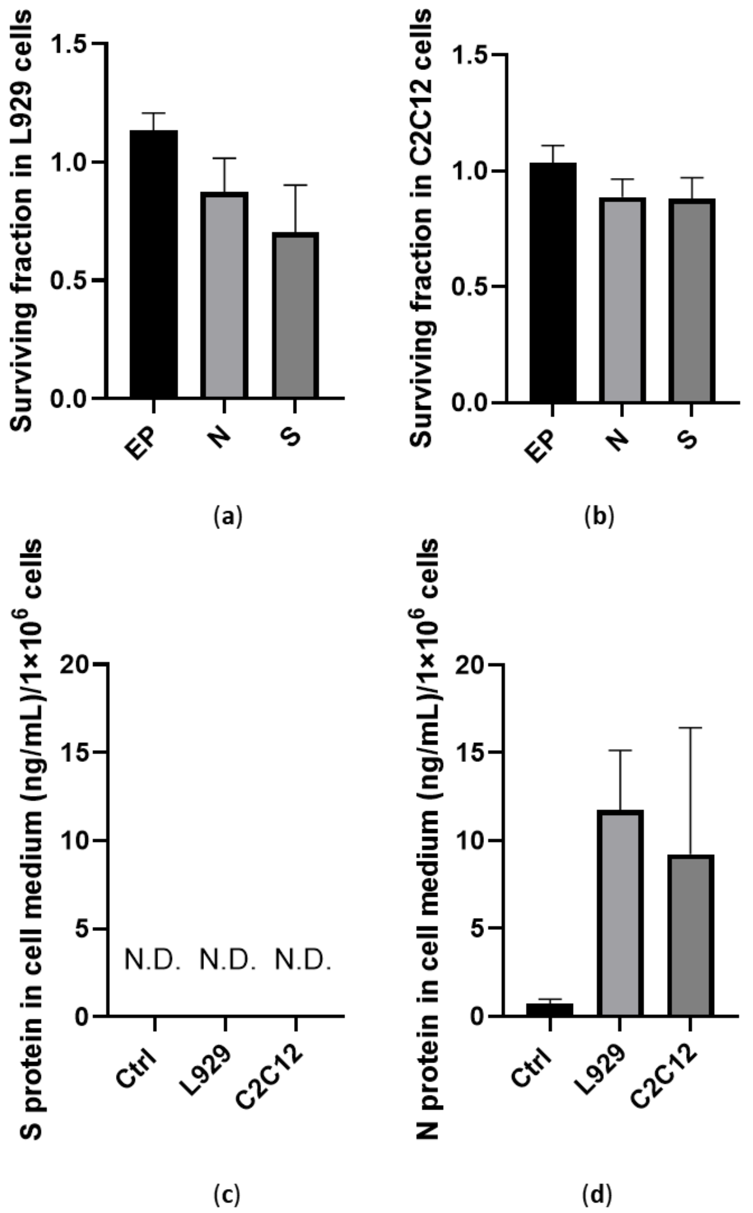

Supplementary Figure S2. In vitro survival and cell-medium S and N protein concentration after vitro GET to L929 fibroblasts and C2C12 myoblasts: (**a**) Survival after GET in L929 cells; (**b**) Survival after GET in C2C12 cells. (**c**) Concertation of S protein in cell medium; (**d**) Concertation of N protein in cell medium. Graph bars represent the mean with standard error of the mean (SEM) of three independent experiments with 3 technical replicates. EP, electroporation alone, GET of pN plasmid, pS, GET of pS plasmid. Ctrl, non-transfected cells; N.D., not detected.

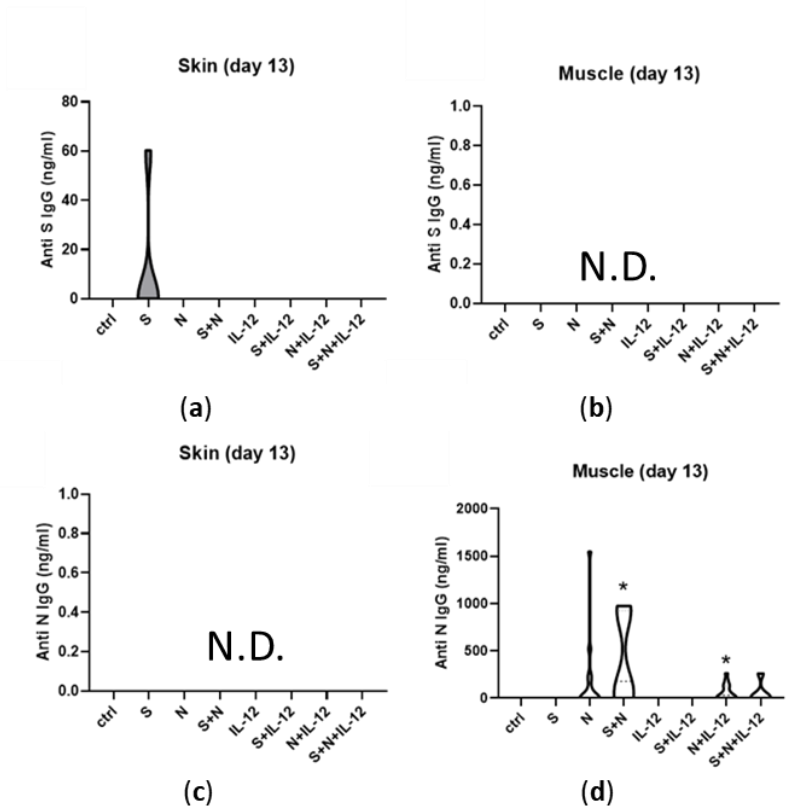

Supplementary Figure S3. Induction of specific IgG antibodies against the transfected S and N antigens in the blood serum of the vaccinated mice 13 days after the first vaccine dose: (**a**) Concentration of anti-S IgG antibodies after skin vaccination; (**b**) Concentration of anti-S IgG antibodies after muscle vaccination; (**c**) Concentration of anti-N IgG antibodies after skin vaccination; (**d**) Concentration of anti-N IgG antibodies after muscle vaccination. Results are presented as violin plots shoving data distribution. 5-10 animals per group. Ctrl, nontreated mice. One-way ANOVA vs Ctrl: *, $P \leq 0.05$. N.D., not detected.
